# Risk of obstructive sleep apnea and quality of sleep among adults with type 2 diabetes mellitus in a sub-Saharan Africa city

**DOI:** 10.11604/pamj.2021.40.264.28310

**Published:** 2021-12-28

**Authors:** Serge Ade, Adebayo Cossi Alassani, Prudence Ablo Wachinou, Audrey Tchemoua Youzeu, Abdoulaye Imorou, Lionelle Fanou, Marius Claude Flatin, Spero Hounkpatin, Gildas Agodokpessi, Anthony David Harries

**Affiliations:** 1 Faculty of Medicine, University of Parakou, Parakou, Benin,; 2 Faculty of Health Sciences, University of Abomey-Calavi, Cotonou, Benin,; 3 Cotonou Army Hospital, Cotonou, Benin,; 4 International Union Against Tuberculosis and Lung Diseases, Paris, France,; 5 Faculty of Infectious and Tropical Diseases, London School of Hygiene and Tropical Medicine, London, UK

**Keywords:** Type 2 diabetes mellitus, obstructive sleep apnea, sleep quality, Benin

## Abstract

**Introduction:**

diabetes mellitus (DM) and Obstructive Sleep Apnea (OSA) are two major and interconnected non-communicable diseases. Both negatively impact on sleep quality. This study aimed to determine among persons with type 2 DM, the proportions at high risk of OSA and of self-reported poor sleep quality along with their associated-factors in Parakou city, Benin.

**Methods:**

this was a cross-sectional prospective study of 100% (n=383) outpatient adults with type 2 DM, conducted between April and August 2019 in the three top centres managing diabetic persons in Parakou city. They were interviewed, examined and investigated using capillary fasting blood glucose tests. The STOP-Bang Questionnaire (SBQ) was used to determine the risk of OSA.

**Results:**

overall, their mean age was 57.37 (11.45) years. They were 61.62% (n=236) females and 38.38% (n=147) males. Sleep duration was insufficient in 26.89% (n=103). Nocturia was reported in 49.35% (n=189). The risk of OSA was high in 14.10% (n=54), intermediate in 24.80% (n=95) and low in 61.10% (n=234). Friedman Position Tongue Grade 3 (Adjusted Odds Ratio, aOR=2.48; 95%CI=1.11 - 5.55; p=0.025) and 4 (aOR=4.65; 95%CI=1.26 - 15.90; p=0.015) were independently associated with a high risk of OSA. The prevalence of reported poor sleep quality was 27.42% (n=105). Female gender (aOR=2.08; 95%CI=1.18-3.83; p=0.014), diabetic foot (aOR=5.07; 95%CI=1.15-23.63; p=0.031), nocturia (aOR=1.96; 95%CI=1.18-3.29; p=0.010), tiredness (aOR=2.77; 95%CI=1.26-6.23; p=0.012) and a high risk of OSA (aOR=3.31; 95%CI=1.28-8.93; p=0.015) were independently associated with a greater risk of reported poor sleep quality.

**Conclusion:**

in Parakou, the proportions of patients with type 2 DM at increased risk of OSA and with poor quality of sleep are relatively high. There is need for better systematic screening of OSA in persons with DM.

## Introduction

Increasing urbanization, sedentary lifestyles, population ageing, changes in feeding, behavioural habits and sleep disruptions have prompted the emergence of non-communicable diseases as a global public health threat. According to the World Health Organization (WHO), non-communicable diseases are nowadays the leading causes of death in the world (1), 85% of these occurring in low- and middle-income countries [1]. Diabetes mellitus (DM) is one of the most common non-communicable diseases, and type 2 diabetes (T2DM) is by far the most frequent, accounting for 90% of all cases. Globally, the prevalence of DM has doubled from 4.7% to 8.5% between 1980 and 2014, and is the seventh leading cause of death [2]. In sub-Saharan Africa, it affects about 19 million people [3]. Similarly, Obstructive Sleep Apnea (OSA), the most common sleep disorder, is another non-communicable disease also on the increase globally. The prevalence of OSA is estimated to have increased from 4% in men and 2% in women in 1993 to 12.5% in men and 5.9% in women in 2016 [4, 5]. The condition, however, remains largely under-diagnosed and under-treated, especially in sub-Saharan Africa.

Several reports in the literature mention numerous complex interrelationships between the two diseases. OSA has been associated with insulin resistance, glucose intolerance and T2DM independently of obesity, with this association increasing with the severity of the sleep disorder [6, 7]. Chronic intermittent hypoxia and sleep disruption resulting from OSA are thought to play a major role in glucose metabolism disturbances [6, 7]. On the other hand, the mechanisms underlining OSA among diabetic patients are less well understood, but may involve an autonomic neuropathy according to some authors (8). Overall, the prevalence of T2DM among those with OSA varies from 5% to 30%, while the prevalence of OSA among persons with diabetes ranges from 58% to 86% [7]. These observations prompted the International Diabetes Federation to recommend in 2008 that patients with one condition should be systematically screened for the other condition [8].

Furthermore, there is evidence of a vicious cycle between both diseases and sleep quality. OSA, by fragmenting the sleep architecture, alters its quality. Among persons with diabetes, there is evidence that poor sleep quality may disrupt glycaemic control [9]. Therefore, diabetic persons with untreated OSA have a high risk of poor sleep quality, chronic hyperglycaemia and its related-complications such as cardiovascular disease. Most studies on these issues between DM, OSA and sleep quality have been conducted in high resources countries. The aim of the present study was to report the experience from a developing setting and to determine among persons with T2DM living in Parakou city in Benin, the numbers and proportions of those with a high risk of OSA and of those who reported a poor sleep quality, as well as their associated factors.

## Methods

**Study design period:** this was a cross-sectional prospective study that was conducted between April 11 and August 5, 2019.

**Setting:** Benin, a developing country located in West Africa, has not been spared by the increasing prevalence of DM worldwide. In 2008, DM prevalence was 2.6% [10]. In 2015, the prevalence of fasting hyperglycaemia (≥6.1 mmol/l) or persons with DM on medication was 12.4% [11]. From a Cotonou study, the economic capital city of the country, the prevalence of OSA found after recording patients with ventilator polygraphy was 53.2% [12].

Parakou, the third major city of the country, is located in Borgou, the region with the highest prevalence of impaired fasting glycaemia in the country at 12.4% [10]. The prevalence of DM in Parakou was 7.9% [10]. The study was carried out in three (2 public and 1 private) of the top-level health centres delivering care to diabetic persons in Parakou city.

**Study population:** the study population was selected from all outpatients with T2DM who were consulted during the study period in one of these three facilities. The inclusion criteria were being aged ≥18 years and giving informed consent. Non-inclusion criteria were: i) not working at night; ii) not having psychiatric disorders; and iii) not being pregnant (for females) at the time of the survey. The sample size was calculated using the Schwartz formula N= ℇ^2^pq/i^2^, with N the sample size, ℇ the 95% confidence level equal to 1.96, p the prevalence of OSA among diabetic persons in Cotonou equal to 53.2% [12], q that is 1 - p being equal to 0.468 and i the accepted margin of error being equal to 5%. The sample size was 383 patients. A systematic sampling of all outpatients with T2DM who met the inclusion criteria was performed.

**Data collection:** a structured interview with the patient was led by a medical student who was at the end of her medical training, using a pre-test and corrected questionnaire. Information on socio-demographic characteristics, DM history, treatment and complications, co-morbidities, lifestyle, sleep patterns, and OSA-suggestive symptoms were sought. Anthropometric parameters, neck and waist circumferences, as well as vital signs were measured. Neck circumference was measured with the head being in a neutral position, halfway between the tip of the chin and the supra-sternal hollow. Waist circumference was measured in a standing position and in gentle exhalation, halfway between the lower costal margin and the iliac crest. These measurements were followed by an Ear Nose Throat (ENT) and oral cavity examination, with a special focus on the presence of any morphological abnormalities. The snoring, tiredness, observed apnea, blood pressure, body mass index, age, neck circumference, and male gender (STOP-Bang) questionnaire (SBQ) was used and a score was derived to assess the risk of OSA [13]. The clinical assessment was completed with a measurement of capillary fasting glycaemia using an Accu-check mobile glucometer at the survey date.

**Diagnostic criteria:** in this study, a high risk of OSA corresponded to any person who was recorded as “high risk” at SBQ. A self-reported poor sleep quality was considered if any patient responded “yes” to the following question: in the last month, do you wake up in the morning at least 4 days in a week with the feeling of being still tired or not having had a restful sleep? Details on other diagnostic criteria used in this study are shown in Table 1[3, 13-18].

**Table 1:** the diagnostic criteria used in this study

Diabetes mellitus (3)	Two venous (or capillary) fasting blood glucose levels ≥ 126 mg / dl (≥ 7 mmol / l); normal fasting blood glucose being between 70 and 110 mg / dl
Poor glycemic control (3)	HbA1c ≥ 7%
OSA probability using STOP-Bang questionnaire (13)	Low OSA risk “Yes” to 0-2 questions
	Intermediate OSA risk: “s” to 3-4 questions
	High OSA risk:
	“Yes” to 5-8 questions or
	“Yes” to at least 2 of the first 4 questions + male sex or
	“Yes” to at least 2 of the first 4 questions + BMI > 35 kg/m^2^
	or
	“Yes” to at least 2 of the first 4 questions + neck circumference (43 cm for men, 41 cm for women)
	Note: details on the different questions are available on http://www.stopbang.ca/osa/screening.php
Excessive daytime sleepiness (14)	Epworth sleepiness scale ≥ 11
Hypertension (15)	Systolic blood pressure ≥ 140 mmHg and/or diastolic blood pressure ≥ 90 mmHg at the time of the investigation or evidence of use of antihypertensive drugs
Nocturia (16)	> 1 urination per night
Nutritional status (17)	Underweight: < 18.50 kg/m^2^
	Normal BMI: 18.50 - 24.99 kg/m^2^
	Pre-obesity: 25.00 - 29.99 kg/m^2^
	Obesity: ≥ 30 kg/m^2^
Abdominal obesity (16)	Waist circumference ≥ 94 cm (man) and 80 cm (woman)
Original Friedman Tongue Position grading (18)	Grade 1: visualization of the entire uvula and tonsils
	Grade 2: visualization of the uvula but not the tonsils.
	Grade 3: visualization of the soft palate but not the uvula
	Grade 4: visualization of the hard palate only

HbA1c=Glycated Haemoglobin; OSA= Obstructive Sleep Apnea; BMI=Body Mass Index

**Statistical analysis:** all data were collected and double entered into EpiData entry Client v.4.4.3.1 (EpiData Association, Odense, Denmark). The data were analysed using the software Rx64 3.6.0. Percentages were determined to describe categorical variables. Means (with standard deviation) and medians (with interquartile range) were used to describe continuous variables appropriately. Factors associated with a high risk of OSA and a poor sleep quality were investigated using univariate analysis followed by a multiple logistic regression. Among potential risk factors, there has been a special focus on morphological ENT abnormalities, the presence of which would increase the risk of OSA. The following factors, namely sex, age, body mass index, hypertension were not included to determine factors associated with high risk of OSA, as they were already part of the SBQ. To investigate potential factors associated with reported poor sleep quality, DM complications, symptoms and the risk of OSA deserved special attention. Levels of significance were set at 5%.

**Ethical considerations:** this study was conducted with the agreement of the Local Ethics Committee for Biomedical Research of the University of Parakou. Informed consent from all persons with T2DM was obtained prior to their inclusion in the study.

## Results

**Participants´ characteristics:** the mean age of the 383 diabetic persons who were enrolled in the study was 57.37 (11.45) years. They were 61.62% (n=236) females and 38.38% (n=147) males. The average follow-up time from the diagnosis date of the DM was 7.45 years (6.22), ranging from 3 months to 38 years. Of the persons with DM, 77.81% (n=298) were treated with oral antidiabetic drugs. The most commonly reported complication was diabetic neuropathy in 34.73% (n=133). Of associated comorbidities, obesity and hypertension were the most commonly diagnosed in 86.95% (n=333) and 62.14% (n=238) respectively (Table 2). On the date of the survey, the mean fasting blood glucose was 1.58 (0.83) g/l, ranging between 0.53 - 5 g/l. Of all the study participants, 8.62% (n=33) had a HbA1c test, of whom 39.39% (n=13) were found to be poorly controlled.

**Table 2:** treatment, comorbidities, complications and lifestyles of persons with type 2 diabetes mellitus, Parakou, Benin: April - August 2019 (N=383)

		n (%)
**Antidiabetes therapy**		
	Oral antidiabetes drugs	298 (77.81)
	Oral antidiabetes drugs + Insulin	80 (20.89)
	Hygiene-dietary measures alone	03 (0.78)
	Insulin alone	02 (0.52)
**Diabetes mellitus related complications**		
	Diabetic neuropathy	133 (34,73)
	Coma	52 (13,58)
	Diabetic retinopathy	20 (05,22)
	Diabetic foot	09 (02,35)
	Cerebrovascular accident	09 (02,35)
	Myocardial infarction	01 (00,26)
**Comorbidities**		
	General obesity (using Body Mass Index)	127 (33.16)
	Abdominal obesity	333 (86.95)
	Hypertension	238 (62.14)
	Heart failure	08 (02,09)
**Lifestyle**		
	Smoking status	
	Current smoker	09 (02.35)
	Ex smoker*	21 (05.48)
	Alcohol consumption	
	Current alcoholic	14 (03.66)
	Ex alcoholic*	20 (05.22)

* Having smoked or drank alcohol more than one year ago

**Sleep duration and events during sleeping:** overall, the mean duration of sleep for all participants was 7.39 (1.39) hours, ranging from 4 to 10.99 hours. Of these persons, the sleep duration was insufficient in 26.89% (n=103), normal in 65.01% (n=249) and too long in 8.09% (n=31). There were 8.09% (n=31) patients who complained of frequent nightmares, and 49.35% (n=189) who complained of more than one nocturnal awakening.

**OSA-related findings:** amongst symptoms suggestive of OSA, nocturia was reported in 49.35% (n=189) persons, excessive daytime sleepiness in 34.73% (n=133), tiredness in 23.24% (n=89), severe daily snoring in 21.15% (n=81), apnea during sleep in 11.49% (n=44) and lack of concentration in 02.61% (n=10). Based on the SBQ, the risk of OSA was high in 14.10% (n=54), intermediate in 24.80% (n=95) and low in 61.10% (n=234). Diabetic persons with a high risk of OSA slept for significantly shorter periods compared with those who had a low or intermediate risk (6,96 ± 1.20 hours vs 7,46 ± 1.41 hours; p=0.011). The frequency of nocturia was comparable in both subgroups (53.70% vs 48.63%; p=0.490). After multivariate analysis, FTP Grade 3 (adjusted Odds Ratio, aOR=2.48; 95%CI=1.11 - 5.55; p=0.025) and 4 (aOR=4.65; 95%CI=1.26 - 15.90; p=0.015) were associated with a high risk of OSA (Table 3).

**Table 3:** morphologic abnormalities associated with a high risk of obstructive sleep apnea among persons with type 2 diabetes mellitus, Parakou, Benin: April-August 2019 (N=383)

	Bivariate analysis			Multi variate analysis		
	cOR	95%CI cOR	P value	aOR	95%CI aOR	P value
Abdominal obesity	2.76	0.85-9.45	0.098	2.39	0.80-10.34	0.167
Deviated nasal pyramid	0.0	-	1			
Asymetric tip	3.08	0.27-34.62	0.337			
Deviated columella	2.05	0.21-20.08	0.529			
**Hypertrophy of the lower horns**			0.899			
Unilateral	0.84	0.34-2.10				
Bilateral	0.84	0.28-2.51				
Long veil	1.99	1.06-3.73	0.033	5.35	0.68-35.70	0.081
Long uvula	1.74	0.91-3.32	0.088	3.36	0.05-2.74	0.269
Macroglossia	0.73	0.31-1.69	0.458	0.66	0.25-1.50	0.353
Micrognatism	0.00	-	0.685	9.91e-06	9.96e-06-9.29e+72	0.990
Retrognatism	0.50	0.06-3.91	0.500	0.65	0.03-3.83	0.696
**Friedman Position Tongue**			0.004			
Grade 2	0.93	0.45-1.91		0.89	0.42-1.87	0.748
Grade 3	3.00	1.38-6.51		2.48	1.11-5.55	0.025
Grade 4	4.42	1.21-14.97		4.65	1.26-15.90	0.015

cOR= Crude Odds Ratio; aOR= Adjusted Odds Ratio; 95%CI= 95% Confidence interval

**Poor sleep quality related findings:** there were 27.42% (n=105) of participants who reported poor sleep quality. After multivariate analysis, factors associated with a greater risk of reported poor sleep quality were being female (aOR=2.08; 95%CI=1.18-3.83; p=0.014), or having a diabetic foot complication (aOR=5.07; 95%CI=1.15-23.63; p=0.031), nocturia (aOR=1.96; 95%CI=1.18-3.29; p=0.010), tiredness (aOR=2.77; 95%CI=1.26-6.23; p=0.012) and a high risk of OSA (aOR=3.31; 95%CI=1.28-8.93; p=0.015) (Table 4).

**Table 4:** factors associated with a reported poor sleep quality among persons with T2DM, Parakou, Benin: April-August 2019 (N=383)

	Bi variate analysis			Multi variate analysis		
	cOR	95%CI cOR	P value	aOR	95%CI aOR	P value
Age ≥ 65 years	0.95	0.58-1.55	0.842			
Sex female	1.52	0.94-2.44	0.085	2.08	1.18-3.83	0.014
Hypertension	1.24	0.77-1.98	0.376			
Diabetic retinopathy	1.14	0.43-3.06	0.790			
Diabetic nephropathy	1.61	0.38-6.84	0.518			
Diabetic neuropathy	2.14	1.35-3.39	0.001	1.60	0.95-2.66	0.074
Diabetic foot	3.43	0.90-13.01	0.056	5.07	1.15-23.63	0.031
Cerebro-vascular accident	0.75	0.15-3.68	0.724			
Nocturia	1.91	1.21-3.02	0.005	1.96	1.18-3.29	0.010
Excessive daytime sleepiness	2.56	1.61-4.06	<0.001	1.01	0.50-1.95	0.986
Tiredness	11.45	6.64-19.75	<0.001	2.77	1.26-6.23	0.012
Severe daily snoring	2.23	1.31-3.78	0.003	0.77	0.33-1.68	0.519
Apnea during sleep	4.28	2.21-8.28	<0.001	1.69	0.73-3.89	0.215
Lack of concentration	4.15	1.15-15.02	0.019	1.86	0.44-8.25	0.394
High risk of OSA at SBQ	4.23	2.23-7.68	<0.001	3.31	1.28-8.93	0.015

cOR= Crude Odds Ratio; aOR= Adjusted Odds Ratio; 95%CI= 95% Confidence interval; OSA = Obstructive Sleep Apnea; SBQ = STOP-Bang Questionnaire

## Discussion

This is one of the few studies from sub-Saharan Africa that has attempted to document sleep quality and the risk of OSA in this vulnerable group of persons with diabetes. With the high prevalence of DM in Borgou region and Parakou city, it was important to document the burden of an associated non-communicable disease, namely OSA, that would potentially increase the risk of cardiovascular complications. Some findings from the study deserve consideration.

First, we were surprised by the reported frequency of OSA symptoms in study participants. Nearly half of the participants suffered from nocturia, one third had excessive daytime sleepiness, and one patient in five reported tiredness and severe snoring. In general health settings, these symptoms are not usually systematically sought for during routine consultation, and they are not always spontaneously listed by patients themselves. Local recommendations have therefore been made that practitioners systematically ask about these symptoms during routine consultation.

Second, nearly one patient in seven (14.10%) was found at high risk of OSA in Parakou city. This prevalence was similar to that reported from Saudi Arabia by Kalakattawi et al. who also applied the same SBQ to determine the risk of OSA [19]. However, when comparing with the other rare clinically-based studies from sub-Saharan Africa, the reported prevalence of OSA was much higher. From two studies in Nigeria that included outpatients in tertiary hospitals, the authors reported a high risk of OSA of 27% and 49.5% in 2014 and 2020 respectively [20, 21]. In another study carried out in Kenya, Sokwalla et al. reported a prevalence of 44% [22]. The differences observed may be explained by the use of another clinical score, the Berlin questionnaire, to determine the risk of OSA [20-22]. In the literature, comparisons between the different clinically validated scores to screen for OSA found a higher sensitivity for the SBQ compared with the Berlin questionnaire, with sensitivity increasing with the severity of the disease [23]. Furthermore, the observed prevalence in Parakou contrasts with published research from developed countries. In these studies, sleep events were more often recorded using polygraphy or polysomnography, regardless of the clinical score; and lack of this technology has been a limitation of the current study. OSA was diagnosed in more than half of diabetic persons using this technology [7, 24]. These findings are consistent with that found in Cotonou (53.2%) after having recorded the sleep pattern of 79 T2DM patients, thanks to an initiative implemented in a private institution [12]. All these reports highlight the importance of acquiring more sophisticated technology for diagnosing non-communicable diseases, even though communicable diseases remain more highly prevalent in sub-Saharan African settings. We need continued advocacy directed at decision-makers with the end goal of being able to systematically record sleeping patterns in diabetic persons in the public sector and therefore allowing appropriate therapies to be more widely available.

Third, we hypothesized that morphological abnormalities, if any, would increase the risk OSA in patients with T2DM. This has been confirmed by the findings, with an increasing risk of OSA in the presence of a FTP Grade 3 or 4, and this is in line with findings in the general population [18]. In practice, such simple physical assessments could be used to identify those who require urgent sleep pattern recording for diagnostic confirmation.

Fourth, up to three diabetic persons in ten (27.42%) reported poor sleep quality. In the literature, approximately one third of diabetic persons suffer from sleep disorders and poor sleep quality [9]. This quite frequent complaint among diabetic persons is probably due to several reasons that include concerns about the disease itself, requirements for self-management, other social life issues, and sleep disorders including OSA which play an important role. In other settings, the situation is even worse. The proportion of diabetic persons with poor sleep quality was nearly double in Kenya at 53% [22]. There is an urgent need to address factors associated with poor sleep quality. One of these, diabetic foot, is a chronic DM complication, the prevention of which requires good glycaemic control. As is also reported from elsewhere, nocturia disturbs the pattern of sleep and this may cause poor sleep quality [9]. In our context, nocturia may represent an OSA symptom, as it may also be a manifestation of poor glycaemic control with resulting polyuria. The comparable proportion of diabetic persons complaining of nocturia in both high and low-risk groups for OSA tends to reinforce the last assertion. This is further supported by the fact that up to 40% of the study participants who had a successful HbA1c test did not have their DM controlled. Another factor independently associated with poor sleep quality was a high risk of OSA at SBQ, through sleep fragmentation. Unsurprisingly, tiredness in the day, resulting from a non-restorative sleep at night, was associated with a greater risk of poor sleep quality. Finally, women with their usual higher workload in our context, including household and professional activities, childcare, and who, additionally, are the first to wake up, more often reported a non-restorative sleep than men.

## Conclusion

Obstructive sleep apnea symptoms were frequent among persons with T2DM in Parakou city, Benin. One in seven participants had a high risk of OSA and this was associated with a FTP grade 3 or 4. The prevalence of poor sleep quality was recorded in three out of ten diabetic patients and was increased in women and in the presence of nocturia, tiredness, a high risk of OSA and diabetic foot. In sub-Saharan Africa cities like Parakou, there is need for better systematic screening of OSA in persons with DM, by making sophisticated confirmatory technology more widely available so that more appropriate treatment can be offered and for improving glycaemic control.

### What is known about this topic

- *Both diabetes mellitus and obstructive sleep apnea are two non-communicable diseases that are interconnected ; and the diagnosis of one of these conditions may prompt the screening of the second one;*
- *Sleep quality is poor for many diabetic patients*.

### What this study adds

- *This study reports one of the rare experiences in assessing the risk of obstructive sleep apnea and the burden of poor sleep among patients with type 2 diabetes mellitus in a developing setting, where communicable diseases continue to receive most attention;*
- *Friedman Position Tongue is a simple oral examination that may contribute to identify those with a high risk of OSA, and therefore might be given priority during sleep recording;*
- *Some obstructive sleep apnea suggestive symptoms such as nocturia and tiredness are found independently associated with poor sleep quality and should be systematically asked diabetic patients during routine consultation*.
